# 730. Ocular Involvement Associated with Rickettsial Infection

**DOI:** 10.1093/ofid/ofab466.927

**Published:** 2021-12-04

**Authors:** Fatma Hammami, Makram Koubaa, Amal Chakroun, Khaoula Rekik, Chakib Marrakchi, Fatma Smaoui, Mounir Ben Jemaa

**Affiliations:** Infectious Diseases Department, Hedi Chaker University Hospital, University of Sfax, Tunisia, Sfax, Sfax, Tunisia

## Abstract

**Background:**

Rickettsiosis, a re-emerging disease, is characterized with a myriad clinical symptoms and various manifestations. Ocular involvement is often misdiagnosed since it’s rarely symptomatic. It especially involves the posterior segment. We aimed to study the clinical, laboratory and therapeutic features of ocular involvement associated with rickettsial infection.

**Methods:**

We encountered a retrospective study including all patients hospitalized for rickettsial infection with ocular involvement in the infectious disease department between 2007 and 2020. The diagnosis was confirmed based on serology (seroconversion) and/or positive polymerase chain reaction for *Rickettsia* in skin biopsy.

**Results:**

A total of 24 patients were included with a mean age of 40±12 years. There were 13 women (54.2%). Sixteen patients sought medical care during the warm months, from June to October (66.6%). The revealing clinical signs were febrile maculopapular skin rash (79.2%), cephalalgia (54.2%) and arthralgia (33.3%). Five patients had visual loss (20.8%). Physical examination revealed conjunctival hyperemia (37.5%) and pathognomonic eschar (29.1%). Laboratory investigations revealed elevated liver enzymes (79.1%), thrombocytopenia (75%) and cholestasis (58.3%). Ocular involvement was unilateral in 14 cases (58.3%). Retinitis was the most common manifestation (70.8%), followed by anterior uveitis (20.8%). Retinal fluorescein angiography, performed in ten cases (41.6%), confirmed retinitis in 8 cases (80%). Both retinal vasculitis and papillary hyperfluorescence were noted in two cases (20%). Patients received doxycycline in 21 cases (87.5%) and fluoroquinolones in three cases (12.5%). The median duration of treatment was 7[6-15] days. The disease evolution was favourable in all cases (100%). No ocular sequelae were noted. Complications were noted in two cases (8.2%) represented by thrombophlebitis (one case) and recurrent seizures (one case).

**Conclusion:**

Systematic fundus examination should be performed in front of suspected rickettsiosis, even in the absence of ocular symptoms and signs. It provides clinical clues to promptly diagnose and treat rickettsiosis.

**Disclosures:**

**All Authors**: No reported disclosures

